# Editorial: Recent developments in clinical biomarkers for cancer prognosis, prediction, treatment, and their clinical utility

**DOI:** 10.3389/fgene.2024.1437556

**Published:** 2024-06-06

**Authors:** Akansha Singh, Arya Ashok, Sanjay Pandey, Qing Lin

**Affiliations:** ^1^ Department of Radiation Oncology, Medical School, University of Texas Southwestern Medical Center, Dallas, TX, United States; ^2^ Tempus Labs, Chicago, IL, United States; ^3^ Department of Radiation Oncology, Albert Einstein College of Medicine, New York, NY, United States; ^4^ Department of Anesthesiology and Critical Care Medicine, School of Medicine, Johns Hopkins Medicine, Baltimore, MD, United States

**Keywords:** genetic biomarkers, ovarian serous cystadenocarcinoma (OV), colorectal cancer (CRC), lung adenocarcinoma, prostate cancer, hepatocellular carcinoma (HCC), cancer prognosis, precision medicine

Biomarkers have become pivotal in cancer research, offering crucial insights into disease progression and treatment response (Parkinson et al., 2014). Despite significant advances in treatment modalities, the prognosis for many cancer patients remains variable, with outcomes ranging from complete remission to aggressive disease progression and mortality. This variability highlights the critical need for precise prognostic tools that can guide clinicians in tailoring treatments and predicting patient outcomes accurately. Recent advancements in genomic and bioinformatic technologies have revolutionized the preparation and utilization of multiple patient databases which has emerged as a powerful tool for biomarker discovery and validation (Pritchard et al., 2022). The collective findings presented in this Research Topic emphasize on the transformative potential of genetic biomarkers in refining cancer prognosis, disease progression, treatment response and discovery of novel biomarkers to help advance personalized medicine approach.

In this Research Topic, inventive research from several groups has uncovered novel prognostic biomarkers and shed light on their role associated with cancer progression using extensive patient databases. Lv et al. conducted a comprehensive analysis across multiple databases for ovarian serous cystadenocarcinoma (OSC) and observed an inverse correlation of Acyl-CoA thoesterase 13 (ACOT13) expression with the stages of OSC progression. Their findings linking ACOT13 expression to immune checkpoint, SIGLEC 15, levels, tumor mutational burden, and chemotherapy, cisplatin, response highlight its potential as both an independent prognostic biomarker and therapeutic target for OSC.

Qiu et al. expanded the utility of TIMP-1, already studied for association with colorectal cancer (Song et al., 2016; Meng et al., 2018), by revealing its implications in drug sensitivity and ferroptosis inhibition, offering avenues for refining treatment strategies in colorectal carcinoma patients. Meanwhile, Zheng et al. broaden the horizon for TOPBP1 interacting checkpoint and replication regulator (TICCR) in lung adenocarcinoma, demonstrating its association with key cancer progression pathways such as cell proliferation, DNA replication, DNA damage repair, cysteine metabolism, *etc.* They further established that their TICRR risk model can accurately predict lung adenocarcinoma patient prognosis.

Prostate cancer is second most common cancer among males worldwide, and its tumor microenvironment (TME) consisting of several non-epithelial cell types surrounding tumor plays a vital role in pathogenesis, progression, and metastasis of prostate cancer (Kang et al., 2022). Nie et al. identified a panel of TME-associated genes and developed a robust risk model consisting of FERMT1, CARD9, IL20RB, MET, and MMP3, providing a valuable tool to be used as an independent prognostic biomarker panel for prostate cancer patients undergoing treatment. Prostate cancer is well known for its dependency on androgen for growth and progression, which is utilized effectively in clinical practices as a therapeutic measure. But a lethal variant, castration-resistant prostate cancer (CRPC) needs novel targeted therapeutic interventions to curb the tumor progression (Teo et al., 2019). Cordier et al., examined the role of calcium-regulated signaling, specifically Transient receptor potential vanilloid subfamily member 6 (TRPV6) calcium channel, in CRPC cell lines. Their investigation into TRPV6 role in chemotaxis, migration, invasion, apoptosis, ferroptosis, drug resistance, as well as extra-cellular matrix (ECM) organization pathways underscores its potential as a therapeutic target, offering new avenues for managing CRPC progression.

In the domain of liquid biopsy, circulation tumor DNA (ctDNA) that are tumor-derived portion of cell-free DNA in patient’s blood, serves as an excellent non-invasive biopsy biomarker pool (Nikanjam et al., 2022). Kumar et al.’s study on ctDNA mutations in HBV-induced hepatocellular carcinoma (HCC) patients unveils the prognostic significance of TP53 and CTNNB1 mutant fraction and frequencies. They showed combined TP53 and CTNNB1 gene mutations were associated with poor survival rate in HBV-HCC patients, suggesting their utility for early detection and monitoring disease progression in HBV-positive high-risk patient cohorts.

Collectively, these findings highlight the transformative potential of genetic biomarkers in refining cancer prognosis, guiding treatment decisions, and advancing personalized medicine approaches for improved patient outcomes. Further research and clinical validation are warranted to fully realize the clinical implications of these promising biomarkers in oncology practice.
